# Isolation and in-vitro characterization of extracellular phytase producing bacterial isolates for potential application in poultry feed

**DOI:** 10.1186/s12866-023-03041-2

**Published:** 2023-10-17

**Authors:** Lubaba Amede Mussa, Diriba Muleta Yadetie, Endeshaw Abatenh Temesgen, Anteneh Tesfaye Tefera, Mesfin Tafesse Gemeda

**Affiliations:** 1 Biotechnology Department, College of Natural and Computational Sciences, Institute of Biotechnology, Addis Ababa University, Addis Ababa, Ethiopia; 2 Biotechnology and Bioprocess Center of Excellence, Addis Ababa Science and Technology University, Addis Ababa, Ethiopia

**Keywords:** Phytase, Rhizosphere soil, Poultry feed

## Abstract

**Background:**

Phytase catalyses the breakdown of complex organic forms of phosphorous into simpler forms by sequential hydrolysis of phosphate ester bonds to liberate the inorganic phosphate. Supplementation of feeds with bacterial phytase therefore could enhance the bioavailability of phosphorus and micronutrients. Hence, the aim of this study was to isolate and characterize phytase producing bacteria from rhizosphere soil, fresh poultry excreta, and cattle shed to evaluate their potential in improving poultry feeds. Phytase producing bacteria were isolated using wheat bran extract medium.

**Results:**

A total of 169 bacterial isolates were purified and screened for phytase activity. Out of these, 36 were confirmed as positive for phytase enzyme activity. The bacterial isolates were identified by cultural, morphological, and biochemical features. The isolates were also identified by using 16 S rRNA gene sequencing. The bacterial isolates (RS1, RS8, RS10 and RS15) were provided with gene bank database accession numbers of MZ407562, MZ407563, MZ407564 and MZ407565 respectively. All isolates increased phytase production when cultured in wheat bran extract medium (pH 6) supplemented with 1% (wt/v) galactose and 1% (wt/v) ammonium sulphate incubated at 50^o^C for 72 h. Proximate composition analysis after supplementation of phytase showed that phytase supplementation improved bioavailability of phosphorus, calcium, potassium and sodium in poultry feed.

**Conclusions:**

Overall, this study showed that the nutritional value of poultry feed can be improved using microbial phytase enzyme which reduces the cost of supplementation with inorganic phosphate.

**Supplementary Information:**

The online version contains supplementary material available at 10.1186/s12866-023-03041-2.

## Introduction

Poultry is an agricultural practice in which domesticated and commercialized types of birds are kept and managed for production of egg and meat for humans. It provides nutritionally beneficial foods that contain high-quality protein accompanied by a low proportion of fat [1–3]. The demand for protein-rich food is gradually increasing with improvements of society’s income and population growth. Poultry products are crucial sources of edible animal protein [3]. To achieve food self-sufficiency and to combat malnutrition in developing countries we must give emphasis for poultry production [2]. Poultry feed is a fundamental need for chicken production and account 60–70% of production costs under intensive production systems [4]. Poultry feed is the uppermost constraint because lack of processing facilities, inconsistent availability and distribution and sub-standard quality of processed feeds during poultry processes in both small holder and large-scale systems [5].

Poultry production is seriously constrained by poor quality poultry nutrition due to large quantity of phosphorus in grain based poultry diet existing in phytate-bound form. As a result, the poultry cannot use bound form of phosphorus due to absence of phytase enzyme in their gastro-intestinal tract [6]. Subsequently, a good amount of phytate in the diet consumed by chickens is excreted resulting in environmental pollution during application of manure to agricultural fields.

This chelated phosphorus in feeds demands the poultry keepers to use high-priced inorganic phosphate in the poultry feed [6]. Phytate also reduces bioavailability of some important cations and is known to reduce digestibility of protein. To overcome these problems during intensive livestock production, food, and feed can be supplemented with phytase for improving phosphorous bioavailability and reducing phosphorous excretion [7].

Phytase is abundantly found in nature and derived from living things. Phytase enzyme from microorganisms can improve poultry feed, making the poultry farm economically useful and minimizing environmental pollution. A few principal phytase producing microorganisms include *Bacillus subtilis, Escherichia coli, Aspergillus niger, Aspergillus oryzae*, *Aspergillus flavus*, *Penicillium sp., Saccharomyces cerevisiae*, and *Schwannoiomy cescastelii* [8]. Microbial origins are predominately promising for a massive production of phytase enzyme at a marketable and profitable scale [9] for its potential in biotechnological applications [10]. In this study, the phytase-producing bacteria were isolated from rhizosphere soils, fresh poultry excreta and cattle shed samples and the enzyme was characterized for poultry feed improvement.

## Materials and methods

### Sample collection

The sampling areas for the current study were selected based on potential poultry and their proximity where the accumulation of phytase producing bacteria was suspected. Samples were collected randomly (X-fashion) sampling method from different sites within the premises of Addis Ababa Science and Technology (AASTU) parameters. Total of 60 samples were gathered. Those are 20 rhizospheric soil samples from common cereal crops (Maize, Wheat, Teff, and Sorghum) and 30 Rhizospheric soil samples from legumes (chickpea, cowpea, field pea, grass pea, faba bean, and lentil) and five fresh poultry excreta samples were obtained from Addis Ababa area. Five cattle shed samples were gathered from Bishoftu area. In this study, samples were aseptically collected by using sterile spatula and plastic bottle. Fresh samples were transported to the microbiology laboratory using ice box and stored at 4^o^C until use.

### Preparation of wheat bran extract medium

Twenty five kilo gram (25 kg) wheat bran was collected from Charily Feed Factory Addis Ababa, Ethiopia. A mass of 1 kg oven dehydrated and pounded wheat bran was suspended in a solution of 10 L of distilled water and a 50 mL of H_2_SO_4_ and after that autoclaved at 121^o^C for 20 min. The fluid fractions were separated by 47 mm pore size filtration and using 1 N of NaOH solution or HCL the pH was adjusted to 6.5. Finally the wheat bran extract was obtained by the removal of water at 50 °C under reduced pressure [11].

### Isolation and screening of phytase producing bacteria

Ten gram of each sample was separately suspended in 90 ml of sterile distilled water and each samples suspension was serially diluted (10^− 1^ up to 10^− 6^ fold). Modified Luria broth (LB) agar medium with wheat bran extract was prepared by using yeast extract 5 g/L, peptone 10 g/L, NaCl 10 g/L, Agar 20 g/L, in 1000 mL of wheat bran extract, final pH adjusted to 7. Each sample suspension (100 μl) was inoculated and spread over the modified Luria broth agar media. Plates were cultured aerobically at 28 °C for 72 h. Suspected isolates were picked up and sub-cultured to purify all isolates [12].

All isolates were screened by re-culturing each of the pure colonies on newly optimized Wheat bran extract agar medium using 0.04%±0.01 (w/v) of (NH4)_2_SO_4,_ 0.02%±0.01 (w/v) of MgSO_4_.7H_2_O, 0.1%±0.01(w/v) of Casein, 0.05%±0.03 (w/v) of KH_2_PO_4_, 0.04%±0.02 (w/v) of K_2_HPO_4_ and 2%±0.04(w/v) of agar with 1000 ml distilled water, pH 6.5. The plates were incubated at 37 °C for 72 h aerobically. Typical colonies which form a clear zone were considered as potential phytase producers. The diameters of halo (Z) and diameters of colony (C) were measured using a caliper. The hydrolysis efficiency of the isolates was determined via the formula Z-C/ C. Isolates above 50% effectiveness were selected and transferred into nutrient agar slant, mixed with 15% glycerol, then stored at 4^o^C for further activities [13].

### Characterization and identification of the selected isolates cultural, morphological, and biochemical characterizations

The four promising phytase producing isolates (RS1, RS8, RS10 and RS15 were subjected to cultural, morphological and various biochemical tests following standard methods [14]. It was further identified up to the genus level according to Bergey’s Manual of Determinative Bacteriology.

### Molecular identification and sequencing

DNA was extracted according to the methods using proteinase K digestion in a lysis buffer via phenol-chloroform extraction method [15]. To extract genomic DNA the putative bacterial isolates, RS1, RS8, RS10 and RS15 were cultured in Wheat Bran Extract broth and incubated at 37°C for 24 hrs in an orbital shaker at 200 rpm. PCR amplification was carried out using 16S rDNAuniversalprimers8f [(Forward primer: 5’AGAGTTTGATCCTGGCTCAG 3’) and 1492r (Reverse primer: 5’ GGTTACCTTGTTACGACTT3’)] in an Eppendorf Thermo cycler. The quality PCR products were sent to Leibiz institute DSMZ (Deutsche Sammlung von Mikroorganismen Zellkulturen) Braunschweig, Germany for sequencing. Sequenced data were edited using Bio Edit Sequence Alignment Editor (Version 7.0.5.3) and Mega x version 10.1.5 software was used for sequence alignments and the construction of phylogenetic trees respectively.

### Crude phytase enzyme extraction

To obtain crude enzyme, the potent isolates were subjected to fermentation in a Wheat Bran Extract Broth Medium containing gram/Litter: (NH_4_)_2_SO_4_ (0.04%±0.01), MgSO_4_.7H_2_O (0.02%±0.01), Casein (0.1%±0.01), KH_2_PO_4_ (0.05%±0.03), and K_2_HPO_4_ (0.04 ± 0.02%). Before sterilization the pH of the medium was adjusted to 6.5. The inoculated medium was incubated on an orbital shaker incubator (200 rpm) at 37^0^ C for 72 h. After 72 h aliquots of 50 mL of the fermented broths from the flasks were taken and centrifuged at 6000 rpm for 30 min at room temperature. The supernatants were used as crude enzyme solution [16].

### Phytase enzyme purification

Partial purification of extracted phytase was done by the use of ammonium sulphate precipitation followed by dialysis. Cell free extract of 50 ml from each isolates was saturated with 20–60% of ammonium sulphate. The contents were incubated overnight and centrifuged at 10,000 rpm for 10 min. Supernatant from each isolate was collected and checked for enzyme activity; pellets were collected for further analysis. The enzyme mixture (pellet) from each isolate was transferred to dialysis tube and immersed in 2mM Tris- HCl buffer at pH-7 at 4 °C for 24 h for dialysis.

### Phytase enzyme activity

Quantification of phytase activity was determined by ferrous sulphate molybdenum blue method for detecting free inorganic phosphate [17]. To obtain cell free supernatant (CFS) from each culture grown in wheat bran medium, 2 ml was centrifuged at 10,000 rpm for 10 min at 4^o^C. From each isolate 0.2 ml of the CFS was mixed with 0.16 ml of substrate solution containing 0.1 M tris-HCl, 2 mM sodium phytate and 2mM CaCl_2_. The mix was incubated at 37 °C for 30 min. Reactions were stopped by the addition of 2 ml of 5% (v/v) of trichloro acetic acid followed by the addition of 2000 μl of colouring reagent. Coloring reagent was freshly prepared by mixing 4 volumes of 1.5% (w/v) ammonium molybdate with 5.5% (v/v) sulfuric acid and 1 volume of 2.7% (w/v) ferrous sulfate solution. Then absorbance was at 700 nm was measured. By using different concentrations of di potassium hydrogen phosphate (K_2_HPO) as a standard, the quantity of inorganic phosphorous released was estimated.

The phytase activities (IU/ml of CFS) were defined as the micromoles of inorganic phosphate liberated in one minute. Phytase activity IU/ml = K*OD *f /S*M*30.

K is slope of standard curve, OD is optical density, f is dilution multiple, S is amount of sample (0.2 ml) used and M is sample weight (0.06).

### Optimization of phytase production

#### Effect of nitrogen

The effect of nitrogen on phytase production was done by addition of 1% (w/w) different nitrogen sources (ammonium nitrate, sodium nitrate, ammonium sulphate, peptone and urea) in different wheat bran extract medium aliquots and selected bacterial isolates fermented at 37^0^ C for 72 h. The fermented broth was collected and centrifuged. Then supernatants were used to estimate phytase activity [16].

#### Effect of carbon

The effect of carbon on phytase production was done by addition of1% (w/w) of different carbon sources (glucose, sucrose, galactose and starch) in different wheat bran extract medium aliquots and the bacterial isolates were fermented at 37^0^ C for 72 h. The fermented broth was collected and centrifuged. Then supernatants were used to estimate phytase activity [16].

#### Effects of incubation period

To optimize incubation period for the maximum phytase production of selected experimental isolates were determined by growing each isolate separately in wheat bran extract medium at 37 °C and pH 6.5. The experiments were carried out individually for 24, 48, 72, and 96 h. The enzyme assays were carried out at each time interval [16].

#### Effects of inoculum size

Phytase producing isolates were inoculated (10% dilution of 24 h old culture) in to Wheat bran extract medium in 250 ml flasks at different inoculum levels (0.1mL, 0.2ml, 0.4ml, 0.6ml, 0.8ml, and 1ml) respectively. The optical density (OD) was measured after 24 h [16].

#### Effects of pH

The effects of pH on phytase production using wheat bran extract medium and selected bacterial isolates was studied by conducting fermentation at pH values of 3, 4, 5, 6, 7, 8, and 9 for 72 h. The fermented broth was collected and centrifuged. Then supernatants of fermented broths after centrifugation were used to estimate phytase activity [16].

#### Effects of temperature

The effects of temperature on phytase production using wheat bran extract medium by bacterial isolates was analysed by setting the fermentation at different temperatures (35, 40, 45, 50, 55 and 60 °C) along with arbitrary control at 37 °C for 72 h.

### Proximate composition of commercial poultry feed before and after addition of phytase enzyme

Both poultry feed from commercial (well-mixed) and poultry feed from commercial with experimental phytase were sent to Bless Agri-food Laboratory Services P.L.C and Ethiopian Public Health Institute (Addis Ababa, Ethiopian) for the proximate composition analysis of crude enzyme, phosphorus, calcium, sodium, and potassium.

### Determination of crude enzyme

The macro Kjeldahl method was implemented to determine the crude enzyme content according to the methods of AOAC [18].

### Mineral determination

Poultry feed used to determine the mineral contents was obtained from Akaki commercial centre, Addis Ababa, Ethiopia. Samples of commercial poultry feed were extracted to analyze minerals (sodium, potassium, calcium and phosphorus) and then estimated as follows. Before and after addition of experimental phytase enzymes, the minerals were determined by atomic absorption spectrometry (SP-AA 5000) for calcium. Flame photometry was applied for sodium and potassium. Spectrophotometric assay was used for the phosphorus. Determination of minerals was performed according to the methods of AOAC [18].

### Data analysis

All the experiments were carried out independently in triplicate. Generated data in laboratory were analyzed in the form of Mean ± Standard deviation (SD) using Statistical Package for Social Sciences (SPSS) version 23 and Microsoft Excel 2010. Duncan’s Multiple Range-Test was used to test differences among means. Statistical significance between mean values was set at (P˂0.05). Bio Edit version 7.0.5.3 and Mega x version 10.1.5 software were used for sequences alignments and phylogentic tree analysis respectively.

## Results

### Screening of purified isolates for phytase production

**Fig. 1:**
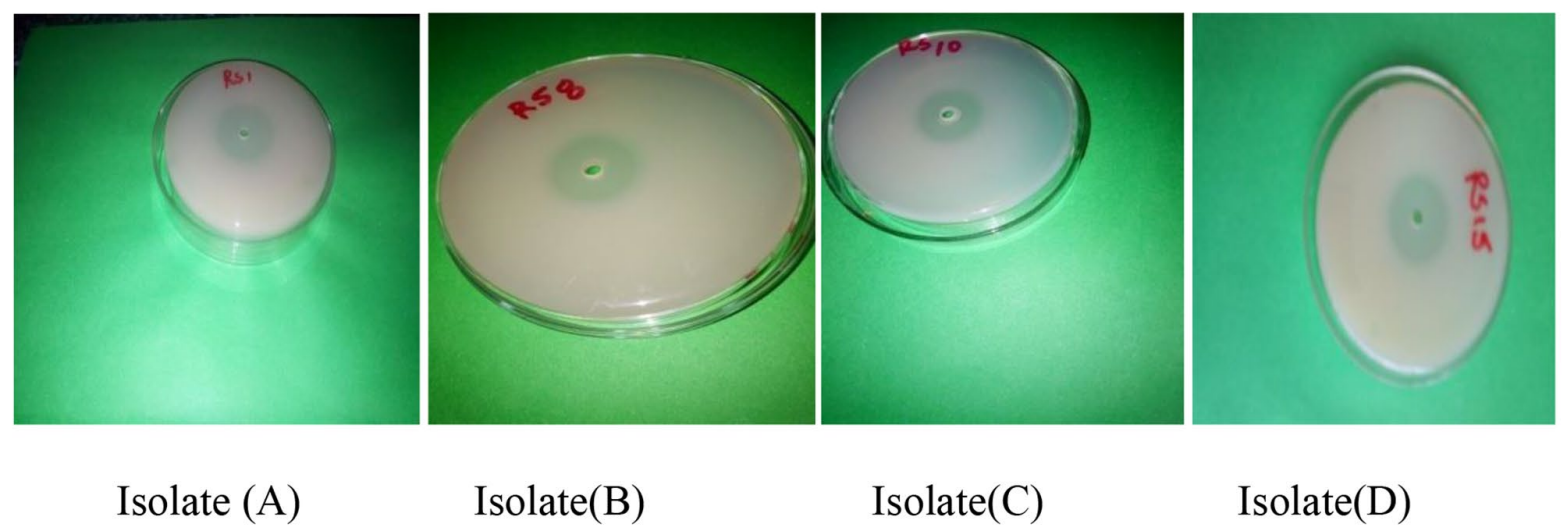
The clear zone hydrolysis efficiency of four phytase producing isolates [**A** (RS1), **C** (RS10), **B** (RS 8), and **D** (RS15)] originated from the same location

From three different sample sources such as rhizosphere soil, cattle shed and fresh poultry excreta sample sources, 169 isolates were selected out of which 36 isolates showed positive for phytase production (Table 1; Fig. 1).

**Table 1:** Total number of phytase producing isolates and their frequency as a percentage (%) of total isolates

No	Sample sources	Number of colonies tested	
		Isolates	Positives
01	Rhizosphere soil samples	72(42.6%)	18(50%)
02	Cattle shed samples	55(32.54%)	10(27.78%)
03	Fresh Poultry excreta samples	42(24.85%)	8(22.22%)
Total		169(100%)	36(100%)

Based on the clear zone of hydrolysis produced by the isolates on wheat bran extract medium we found rings from the least 10 mm to the largest 32 mm. From 36 positive isolates, 16 isolates produced large clear zone of hydrolysis (≥10 mm) and were tested further. From the 16 isolates, 4 bacterial isolates producing clear zones (30 up to 32 mm) were chosen for this study (Table 2).

**Table 2:** The efficiency of phytase producing bacterial isolates selected from rhizospheric soil legumes using wheat bran agar media

No	Suspected bacterial Isolates	Halo diameter (mm)	Colony diameter (mm)	Efficiency of hydrolysis Z-C/C (%)
01	RS 1	31	5	52%
02	RS 8	32	5	54%
03	RS 10	30	5	50%
04	RS 15	30	5	50%

Bacterial isolates A (RS1) and C (RS10) were from grass pea and faba bean field respectively. Whereas as isolate B (RS 8) and D (RS15) were cultured from Lentil soil using wheat bran extract agar media (Fig. 1) below.

### Characterization and identification of the selected phytase producing bacterial isolates

Cultural, morphological and biochemical test characteristics for the four bacterial isolates (RS1, RS8, RS10 and RS15) are shown in Table 3.

**Table 3:** Cultural, morphological and biochemical test characteristics of the selected isolates

No	Cultural characteristics	RS1	RS8	RS10	RS15
01	Colony size	Small	Small	Medium	Medium
02	Shape	Circular	Circular	Irregular	Irregular
03	Color	White	White	Blond	Blond
04	Margin	Entire	Entire	Filaments	Undulate
05	Elevation	Flat fish	Flat fish	Flat	Flat
	**Morphology characters**				
06	Gram Staining	Negative	Negative	Negative	Negative,
07	Cell- shape	Rod	Rod	Rod	Rod
08	Cell arrangement	*Diplobacillus*	*Streptobacillus*	*Cocobacillus*	*Bacillus*
	**Biochemical tests**				
09	Catalase production		+	+	+
10	Casein hydrolysis				
11	Urease production		+		
12	Starch hydrolysis				
13	Hydrogen sulphide production				
14	Carbohydrate fermentation				
15	Suggested genus	*Enterobactersp*	*Klebsiella pneumonia sp*	*Pseudomonas sp*	*Escherichia coli sp*

Key: (-) indicates negative test and (+) indicates positive test

### Polymerase chain reaction (PCR) amplification 16 S rRNA from the selected isolates

**Fig. 2:**
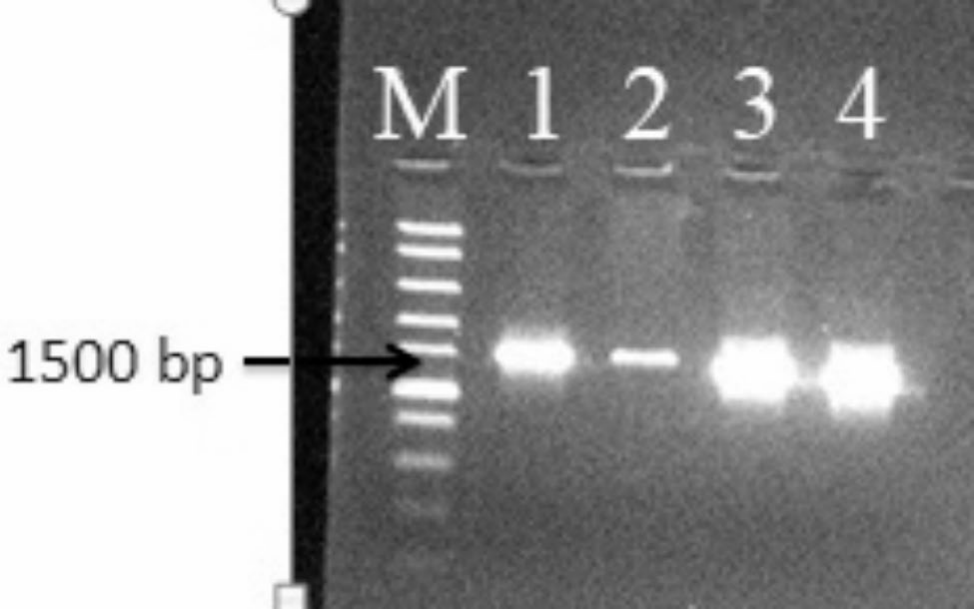
Amplified 16 S rDNA of the four bacterial isolates and the control run in 0.8% (w/v) of agarose gel: M- 1 kb DNA ladder, 1–4 showed the 1500 bp 16 S rRNA genes of RS1, RS8, RS10 and RS 15 respectively

Amplification of the 16s rRNA genes from the genomic DNA of bacterial isolates RS1, RS8, RS10 and RS15 with bacterial specific universal primers for 16 S rRNA generated 1500 bp of PCR product (Fig. 2).

### Identification based on 16 S rRNA sequences

**Fig. 3:**
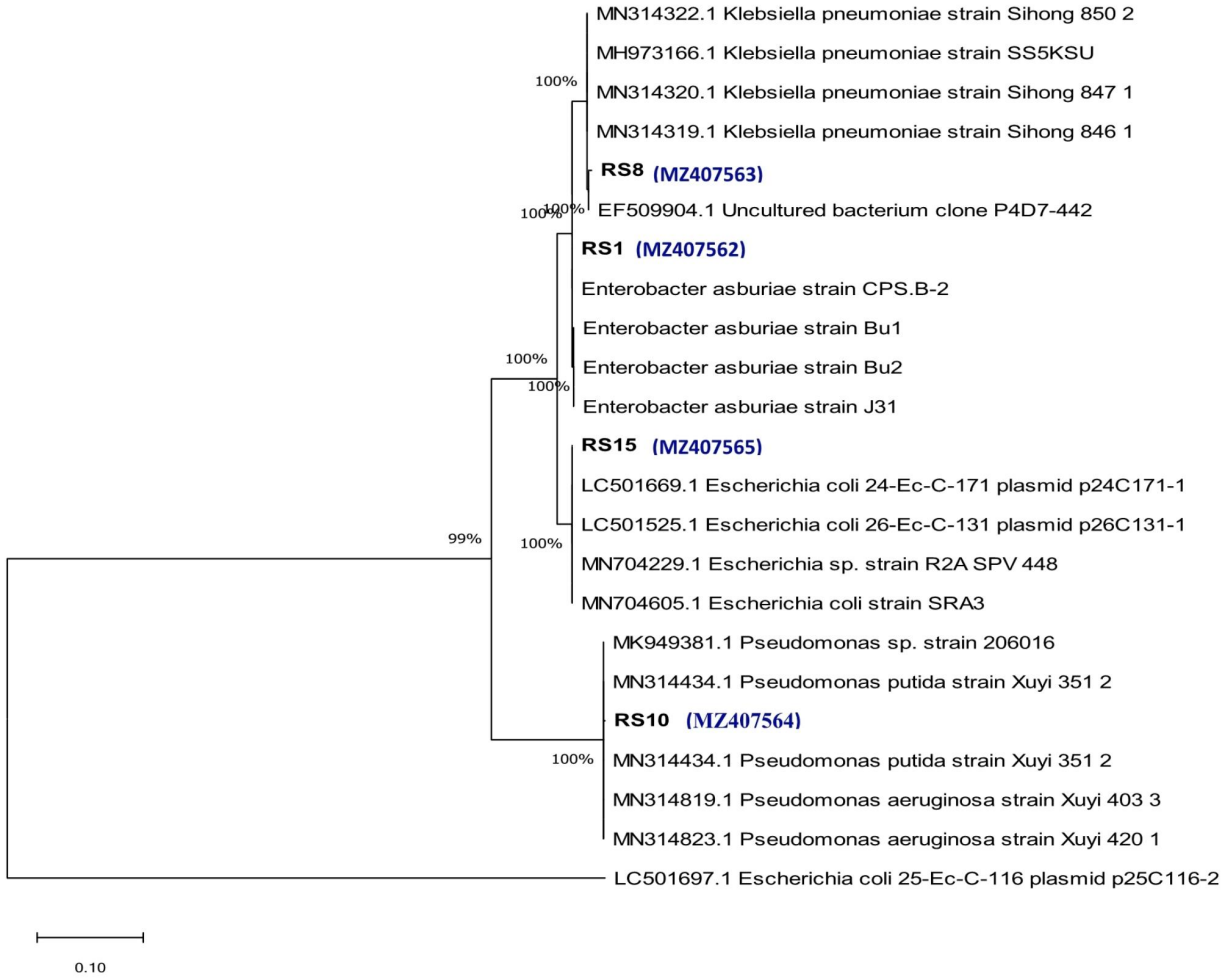
Phylogenetic tree of selected bacterial isolates based on 16 S rRNA sequences

The sequenced 16s rRNA amplicons of the four pure selected bacterial isolates RS1, RS8, RS10 and RS15 were submitted to NCBI GenBank database and assigned accession numbers as MZ407562, MZ407563, MZ407564 and MZ407565 respectively. The 16 S rRNA partial gene sequences were analysed using NCBI BLAST program, phylogenetic tree was constructed, and the nucleotide homology based dendrogram is indicated (Fig. 3).

Phylogenetic analysis of the bacterial strain RS1 (MZ407562), RS8 (MZ407563), RS10 (MZ407564) and RS15 (MZ407565) was made in comparison with other closely related bacterial strains retrieved from NCBI Gen Bank. Similarity and homology of neighbouring sequences has been shown by the robustness tree determined by the analysis of 1000 time’s bootstrap values between them.

### Crude and purified phytase enzyme activity

During sub-merged fermentation, isolated bacteria were producing high amounts of total crude enzyme, crude enzyme activity and partially purified enzyme activities (Table 4).

**Table 4:** Levels of total crude enzyme, crude enzyme activity and partially purified phytase activities from isolated species (Mean ± Standard deviation)

No	Name of bacterial species	Total Crude enzyme (g/100 g)	Crude enzyme activity (IU/ml)	Partially Purified enzyme activity (IU/ml)
01	*Enterobacter asburiae* (RS1)	1.00 ± 0.01	0.061 ± 0.03	0.029 ± 0.01
02	*Klebsiella pneumonia* (RS8)	0.72 ± 0.04	0.054 ± 0.02	0.027 ± 0.03
03	*Pseudomonas aeruginosae* (RS10)	1.09 ± 0.03	0.049 ± 0.04	0.019 ± 0.02
04	*Escherichia coli* (RS15)	0.78 ± 0.1	0.071 ± 0.6	0.058 ± 0.4

### Optimization for phytase production

#### Effect of nitrogen

Bacterial isolates (RS1, RS8, RS10, and RS15) were tested for the effect of different nitrogen source on phytase production during submerged fermentation (Supplementary Fig. S1).

In this study, we examined the effect of ammonium sulphate, ammonium nitrate, peptone, sodium nitrate and urea on phytase production. Bacterial isolates RS1 and RS8 were producing maximum phytase 0.046IU/ml and 0.056IU/ml respectively. Better enzyme production was recorded at 1% of ammonium sulphate at 37 °C, pH 6.5, and 200 rpm and at 72 h. From several nitrogen sources ammonium sulphate treatment supported the highest enzyme activity (IU/ml) in RS1 and RS8.

#### Effect of carbon

Bacterial isolates (RS1, RS8, RS10, and RS15) were tested for the effect of different carbon sources at 1% during submerged fermentation (Supplementary Fig. S2).

The effect of glucose, sucrose, galactose and starch on phytase production for all isolates (RS1, RS8, RS10, and RS15) indicated that the maximum phytase production 0.027, 0.042, 0.023 and 0.026 IU/ml were recorded at 1% galactose respectively at 72 h, at 200 rpm, at 37 °C and pH at 6.5. The maximum phytase activity was record at 72 h, at 200 rpm, at 37 °C and pH at 6.5. However, phytase activity was reduced at 96 h. The galactose treatment improved the enzyme activity.

#### Effects of incubation time

Bacterial isolates (RS1, RS8, RS10, and RS15) were tested for the effect of different incubation times on phytase production (Supplementary Fig. S3). All suspected isolates were optimal at 72 h incubation time with the uppermost enzyme activity.

#### Effects of inoculum size

The effect of the size of inoculums (100 μl up to 1000 μl v/v) on the production of phytase by selected four isolates are shown in supplementary Fig. S4. All tested bacterial isolates were found to be optimal at 800 μl inoculum based on the highest enzyme activity (IU/ml).

#### Stability of phytase at different pH

The effects of different pH values 3 up to 9 on the production of phytase of four selected bacterial isolates are shown in supplementary Fig. S5. All bacterial isolates were found to be optimal at pH 6 with the highest enzyme activity.

#### Stability of phytase at different temperatures

Stability of phytase at different temperatures of the enzyme production by the four selected bacterial isolates was observed (Supplementary Fig. S6). All bacterial isolates were optimal at value 55^o^C with the highest enzyme activity.

### Proximate composition of poultry feed before and after addition of experimental phytase enzyme

The results of proximate analysis of commercial poultry feed (PF) as control and poultry feed with phytase enzymes (E1, E2, E3 and E4) as treatments are shown in Table 5. The pH of the poultry feed was 6.5. In this study, phytase from the bacteria was mixed with the poultry feed with a short time to gain activity. When phytase from the bacteria were mixed with the poultry feed, its activity was lost quickly. The result of proximate compositions analysis of poultry feed with phytase enzymes (E1, E2, E3 and E4 extracted from selected bacterial isolates RS1, RS2, RS3 and RS4 respectively) showed an improvement in the bioavailability of phosphorus, calcium, potassium and sodium (Table 5).

**Table 5:** Analysis of poultry feed (Mean ± Standard Error of the Mean)

Parameters	Before (only feed)	After [feed with phytase (500u/kg)]				SEM	Sig.
	PF	E1	E2	E3	E4		
P mg/100 g	80.15^b^	81.75^c^	80.85^b^	82.13^c^	83.85^a^	0.13	*
Ca mg/100 g	142^a^	497^b^	186^c^	271^c^	244^a^	0.16	*
K mg/100 g	514.99^b^	537.25^a^	531.24^b^	537.73^c^	533.59^a^	0.22	*
Na mg/100 g	317.78^a^	326.41^d^	323.22^a^	319.8^c^	322.19^c^	0.13	*

Key: PF (Poultry feed), E1 (Enzyme one from RS1), E2 (Enzyme two from RS8), E3 (Enzyme three from RS10), and E4 (Enzyme four from RS15). SEM = Standard Error of the Mean; Sig. = Significance level; * = Significant at P ≤ 0.05

^a, b, c, d^: Means in the same row with different superscripts are significantly different (P ≤ 0.05)

The addition of phytase from the bacterial isolates could improve the levels of ionic nutrients found in the commercial poultry feed. The proximity analysis of poultry feed with phytase enzymes infers high improvement in the bioavailability of phosphorus (83.85^a^), calcium (497^b^), potassium (537.73^c^) and sodium (326.41^d^) were from RS15, RS8, RS10 and RS1 respectively. The statistical analysis showed that more than one enzyme additive to poultry feed increased the mean nutrient levels significantly compared to the untreated feed (Table 5).

## Discussion

Phytase producing bacterial isolates were selected based on their activity of phytase production and their clear zone hydrolysis around the colony. These isolates showed a range of halo from 10 to 31 mm size on wheat bran extract agar plates. This study was in agreement with earlier studies that reported clear zone of phytate hydrolysis of > 6 mm [19].

Microbial identification was conducted using 16 S rRNA partial gene sequences as unique genetic fingerprinting of bacterial isolate [6]. Nitrogen sources are the most common to effectively induce phytase enzyme production by bacterial species [20]. The reason for phytase production become decreased, might be depletion of nutrients, death phase of the life form and accumulation of by-products (such as toxins, inhibitors and proteolysis activities in the medium) [21].

The effect of inoculum size of the selected phytase producing bacterial isolates (RS8, RS1, RS10 and RS15) were clarified; maximum phytase production was recorded in the inoculum size at 800 μl (v/v) at 72 h, at 200 rpm, at 37 °C and pH at 6.5. In this study isolates grew on wheat bran extract culture media with different inoculum sizes. Isolates (RS1, RS8, RS10 and RS15) had the maximum phytase productions 0.046, 0.048, 0.04 and 0.044 IU/ml respectively. The result is expected due to the fact that at low inoculum level, growth of the organism might be reduced resulting in insufficient biomass and prolonged time for the organism to enter the stationary phase. This in turn increases the time needed for consuming the substrate and synthesizing the desired product (enzyme). On the other hand, at high concentration (size) of inoculums, the bacteria grew rapidly and the nutrients present in the media became insufficient to support the increased number of bacteria [16]. In addition, higher inoculum sizes may result in a rapid over population of the bacteria and may cause problem of aeration, rapid pH change of the medium [16]. This may affect the phytase activity of the bacterial isolates.

The effect of different pH values from 3 up to 9 on the production of phytase of four selected bacterial isolates was shown. For all isolates (RS1, RS8, RS10 and RS15) the phytase activity increased and reached the maximum at pH 6 (0.046, 0.049, 0.049, and 0.047IU/ml) respectively at 72 h, at 200 rpm, at 37 °C and pH at 6.5. At pH 9, phytase production slowed down relative to the rest of the pH values tested.

The effect of different temperature values (30, 37, 40, 50 and 55 °C) on the phytase production was evaluated at 72 h, 200 rpm, and pH of 6.5. For RS1, RS8, RS10 and RS15 isolates the optimum temperature for phytase activity was recorded at 50 °C. During this study, the phytase activity was reduced at 55 °C.

Proximate composition analysis of phosphorus, calcium, potassium and sodium before treating the poultry feed was used as control. Poultry feed mixed with phytase enzymes from E1, E2, E3 and E4 were considered as experimental treatments. Phytase as a supplement to the poultry feed was increased when compared to the control. The proximate composition analysis after supplementation of phytase with commercial poultry feed was significant to release phytate-bound phosphorus. Interestingly, improvement of bioavailability of phosphorus, calcium, potassium and sodium was observed (Table 5). Phytase when added to poultry feeds increased the proportion of feed ingredients available to the birds and increases feed efficiency [22]. It is possible that phytase could improve feed efficiency without affecting feed intake [23].

## Conclusion

Overall, phytase was obtained from RS1, RS8, RS10 and RS15 isolates. The experimental phytase enzyme preparations were evaluated in vitro and tested for breaking down of phytate bound. The four isolates are powerful sources of phytate-degrading enzyme and suitable for the purpose of improving poultry feeds.

The optimum phytase production of the selected bacterial isolate was studied with different parameters. In this study, the maximum phytase activity (0.042IU/ml from RS8 at 1% galactose, 0.056IU/ml from RS8 at 1% ammonium sulphate, 0.038 IU/ml from RS15 at 50^o^C, 0.049 IU/ml from RS8 and RS10 at pH 6, 0.04 IU/ml from RS1 and RS15 at 72 h and 0.048 IU/ml from RS8 at 800 μl obtained under cultivation conditions agitated at 200 rpm, 37^o^C, and pH 6.5 after 72 h of incubation time. Finally, these bacterial isolates could be potential candidates for the production of phytase and applicable in poultry feed industries.

### Recommendations

Further study should be conducted for different factors like agitation, carbon and nitrogen source either separating or mixing together for the phytase production. Further work is needed regarding optimization methods such as factorial design since combining multiple factors could give best interactions of the factors for maximum phytase production. Lastly, improvements (such as classical, mutation and/or genetic modification) of the phytase producing bacterial isolates should be performed to scale up poultry production at industrial level.

### Electronic supplementary material

Below is the link to the electronic supplementary material.

**Supplementary Material 1: Fig. S1** The effect of different nitrogen sources on phytase production. **Fig. S2** The effect of different carbon sources on phytase production. **Fig. S3** The effect of incubation time on phytase production. **Fig. S4** The effect of different inoculum size on phytase production. **Fig. S5** The stability of different pH on phytase production. **Fig. S6** Stability of phytase enzyme at different temperatures of the enzyme production by the bacterial isolates

## Data Availability

The datasets used and/or analyzed during the current study are available from the corresponding author and first author upon reasonable request. The sequence data of four bacterial isolates generated in this study were deposited to NCBI database with accession numbers of MZ407562, MZ407563, MZ407564 and MZ407565.

## Abbreviations

16S rDNA: 16 S ribosomal DNA
16S rRNA: 16 S ribosomal RNA
AAM: Acetone-acid-molybdate
AASTU: Addis Ababa Sciences and Technology University
LB: Luria broth
NCBI: The national center for biotechnology information
OD: Optical Density
OVAT: One Variable at a Time
rpm: Revolutions per minute
spp.: Species
