# Supplementary material for: Isolation and in-vitro characterization of extracellular phytase producing bacterial isolates for potential application in poultry feed
Source: BMC Microbiol. 2023 Oct 17;23:296. doi: 10.1186/s12866-023-03041-2 (PMC10580623; doi:10.1186/s12866-023-03041-2)
Supplement: Supplementary file 1 — Supplementary Material 1: Fig. S1 The effect of different nitrogen sources on phytase production. Fig. S2 The effect of different carbon sources on phytase production. Fig. S3 The effect of incubation time on phytase production. Fig. S4 The effect of different inoculum size on phytase production. Fig. S5 The stability of different pH on phytase production. Fig. S6 Stability of phytase enzyme at different temperatures of the enzyme production by the bacterial isolates [file 12866_2023_3041_MOESM1_ESM.docx]

**Supplementary file**

**Fig. S1** The effect of different nitrogen sources on phytase production

**Fig. S2** The effect of different carbon sources on phytase production

**Fig. S3** The effect of incubation time on phytase production

**Fig. S4** The effect of different inoculum size on phytase production

**Fig. S5** The stability of different pH on phytase production

**Fig. S6** Stability of phytase enzyme at different temperatures of the enzyme production by the bacterial isolates
